# Circulating Levels of Brain-Enriched MicroRNAs Correlate with Neuron Specific Enolase after Cardiac Arrest—A Substudy of the Target Temperature Management Trial †

**DOI:** 10.3390/ijms21124353

**Published:** 2020-06-19

**Authors:** Francesca Maria Stefanizzi, Niklas Nielsen, Lu Zhang, Josef Dankiewicz, Pascal Stammet, Patrik Gilje, David Erlinge, Christian Hassager, Matthew P. Wise, Michael Kuiper, Hans Friberg, Yvan Devaux, Antonio Salgado-Somoza

**Affiliations:** 1 Cardiovascular Research Unit, Department of Population Health, Luxembourg Institute of Health, L-1445 Strassen, Luxembourg; francescamaria.stefanizzi@lih.lu (F.M.S.); lu.zhang@lih.lu (L.Z.); antonio.salgadosomoza@lih.lu (A.S.-S.); 2 Department of Anesthesia and Intensive Care, Clinical Sciences, Lund University and Helsingborg Hospital, SE-25187 Lund, Sweden; niklas.nielsen@med.lu.se; 3 Department of Cardiology, Clinical Sciences, Lund University and Skane University Hospital, SE-221 85 Lund, Sweden; josef.dankiewicz@gmail.com (J.D.); patrik.gilje@med.lu.se (P.G.); david.erlinge@gmail.com (D.E.); 4 Medical and Health Department, National Fire and Rescue Corps, L-2557 Luxembourg, Luxembourg; pascal.stammet@secours.etat.lu; 5 Department of Cardiology B, The Heart Centre, Rigshospitalet University Hospital, 2100 Copenhagen, Denmark; christian.hassager@regionh.dk; 6 Department of Intensive Care, University Hospital of Wales, Cardiff CF144XW, UK; mattwise@doctors.org.uk; 7 Department of Intensive Care, Leeuwarden Medical Centrum, 8934 Leeuwarden, The Netherlands; mi.kuiper@wxs.nl; 8 Department of Anesthesia and Intensive Care, Clinical Sciences, Lund University and Skane University Hospital, SE-221 85 Lund, Sweden; hans.a.friberg@gmail.com

**Keywords:** microRNAs, biomarker, prognostic, cardiac arrest, neurological function

## Abstract

Outcome prognostication after cardiac arrest (CA) is challenging. Current multimodal prediction approaches would benefit from new biomarkers. MicroRNAs constitute a novel class of disease markers and circulating levels of brain-enriched ones have been associated with outcome after CA. To determine whether these levels reflect the extent of brain damage in CA patients, we assessed their correlation with neuron-specific enolase (NSE), a marker of brain damage. Blood samples taken 48 h after return of spontaneous circulation from two groups of patients from the Targeted Temperature Management trial were used. Patients were grouped depending on their neurological outcome at six months. Circulating levels of microRNAs were assessed by sequencing. NSE was measured at the same time-point. Among the 673 microRNAs detected, brain-enriched miR9-3p, miR124-3p and miR129-5p positively correlated with NSE levels (all *p* < 0.001). Interestingly, these correlations were absent when only the good outcome group was analyzed (*p* > 0.5). Moreover, these correlations were unaffected by demographic and clinical characteristics. All three microRNAs predicted neurological outcome at 6 months. Circulating levels of brain-enriched microRNAs are correlated with NSE levels and hence can reflect the extent of brain injury in patients after CA. This observation strengthens the potential of brain-enriched microRNAs to aid in outcome prognostication after CA.

## 1. Introduction

Cardiac arrest (CA) is the third leading cause of death in industrialised countries. Prediction of neurological outcome of comatose patients after CA is fundamental for optimal treatment for each patient. Currently, multimodal approaches are recommended to prognosticate outcome in these patients [1]. These strategies and their associated predictive models include several tests such as brain computed tomography, electroencephalogram, somatosensory-evoked potentials, pupil light reflex and the measurement of serum neuron-specific enolase (NSE) [2].

NSE has been widely studied as a prognostic biomarker after CA [3], as it is easily detectable and independent from the effect of sedatives [4]. However, the use of this protein biomarker has a number of limitations. First, it is accepted as marker of other non-brain diseases such as small cell lung cancer, renal cell carcinoma and several other syndromes [5], thus, increasing the risk of false positives results. Second, the lack of standardised protocol for assaying NSE levels [6,7] prevents the use of a single threshold for outcome prediction. Third, since this protein is released from the brain into the bloodstream after injury, the permeability of the brain blood barrier—variable across a spectrum of brain injury—affects the circulating levels of NSE reflecting the extent of brain damage [8], resulting in a lower sensitivity of NSE. Therefore, there is a need to discover new biomarkers to be combined with existing prognostication strategies [9].

MicroRNAs (miRNAs—short single stranded non coding RNAs) are increasingly recognised as potential disease markers. They can be found in different body fluids circulating either freely, bound to proteins, or packaged into microvesicles where they are protected against RNase degradation [10,11]. It is noteworthy, miRNAs can be released at an earlier stage than proteins during a pathological process and their expression levels are easily detectable using different techniques such as quantitative PCR, microarrays and high-throughput sequencing, with high specificity and sensitivity [12]. We and others have reported the potential of miRNAs, such as brain-enriched miR-124-3p, to aid in outcome prognostication after out of hospital cardiac arrest [13,14]. Combined approaches using several miRNAs appeared to provide an incremental predictive value [15]. Although it has been reported that the brain blood barrier is disrupted within the first 24 h after return of spontaneous circulation after cardiac arrest [16], allowing the release of miRNAs from the brain into the blood, it is still unclear whether circulating levels of miRNAs reflect the extent of brain damage. This is a prerequisite to the value of miRNAs as prognostic biomarkers after cardiac arrest.

To address this, we tested a potential correlation circulating levels of miRNAs and NSE measured 48 h after return of spontaneous circulation (ROSC).

## 2. Results

Two groups of 25 cardiac arrest patients with either a good (CPC 1) or a poor (CPC 5) neurological outcome at 6 months were enrolled in this study. Each group had an equal percentage of males and females (84% and 16%, respectively). Patients with a poor neurological outcome were older, had more frequent arrhythmias, a longer time from cardiac arrest to ROSC, higher levels of brain natriuretic peptide, creatinine, procalcitonin, S100 and NSE (Table 1).

Small RNA sequencing was performed in plasma samples drawn 48 h after ROSC. An average of 18.5 million reads per sample was obtained. Data are available at the Gene Expression Omnibus under the reference GSE74198. After filtering out miRNAs expressed with less than five counts in less than 12 samples of at least one of the two groups of patients, a total of 673 miRNAs remained and were considered for subsequent analysis. We investigated the correlations between these miRNAs and demographic and clinical variables including age and sex, comorbidities, arrest conditions and laboratory measurements (i.e., all variables contained in Table 1), first in the entire group of 50 patients, and then separately in the good and poor outcome groups. A threshold of 0.6 was used to select significant correlations between miRNAs and continuous clinical variables, and an adjusted *p*-value below 0.05 was used for categorical variables.

**Figure 1:**
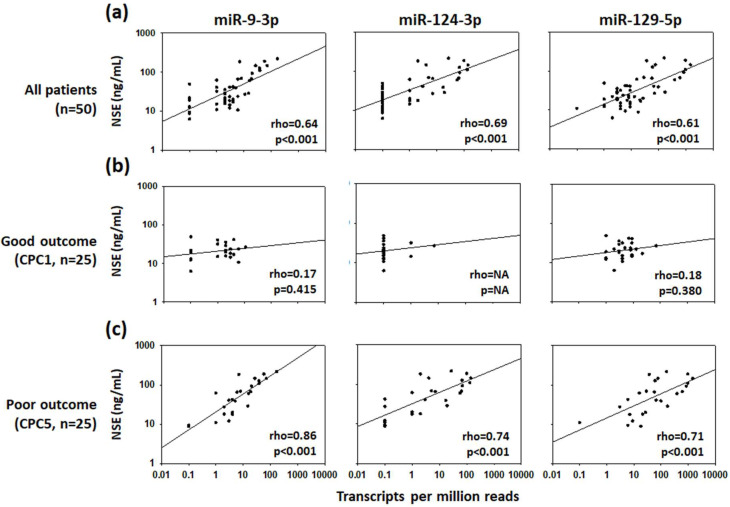
Correlations between miRNAs and neuron-specific enolase (NSE). Scatter plot and linear regression lines show the correlation between NSE and levels of miR9-3p, miR124-3p, and miR129-5p, (**a**) measured 48 h after ROSC in 50 patients, (**b**) 25 patients with good neurological outcome (CPC 1) and (**c**) 25 patients with poor neurological outcome or died (CPC 5) at 6 months after cardiac arrest. Spearman correlation coefficients (rho) and *p*-values are indicated. NA = Not applicable as only few patients have miRNA values above the level of detection.

In all patients, a positive correlation was found between the expression levels of three miRNAs (miR9-3p, miR124-3p and miR129-5p) and NSE measurements at 48 h after ROSC with correlation coefficients of 0.64, 0.69 and 0.61, respectively (Figure 1a). Among the 673 detected miRNAs, these three miRNAs were the only ones correlated with NSE with a correlation coefficient above 0.6. When the neurological outcome at 6 months was taken into account, the correlation coefficients between the three miRNAs and NSE were far below 0.60 in the good outcome group (Figure 1b). Notably, in the poor outcome group, the correlations between the three miRNAs and NSE were even stronger than in all patients, reaching correlation coefficients of 0.86 for miR9-3p, 0.74 for miR124-3p and 0.71 for miR129-5p (all with *p* < 0.001; Figure 1c).

In addition, miR124-3p and miR129-5p were correlated with the time from cardiac arrest to ROSC in the poor outcome group (rho = 0.66, *p* = 3 × 10^−4^; rho = 0.67, *p* = 3 × 10^−4^; respectively). In the good outcome group, miR124-3p was detectable only in four patients with a number of reads ranging from one to seven compared to the CPC 5 group where the number of reads raised until 140 per patient in a total of 19 patients. Also in the good outcome, miR129-5p was not correlated with the time from cardiac arrest to ROSC (rho = −0.04, *p* = 0.863). MiR9-3p was not correlated with the time from cardiac arrest to ROSC, either in the poor outcome group (rho = 0.53, *p* = 0.006) or in the good outcome group (rho = −0.19, *p* = 0.355). Remarkably, no significant correlation was found between the three miRNAs and the comorbidities shown in Table 1, either in the fifty patients or in the separate outcome groups (all adjusted *p*-values above 0.05).

**Figure 2:**
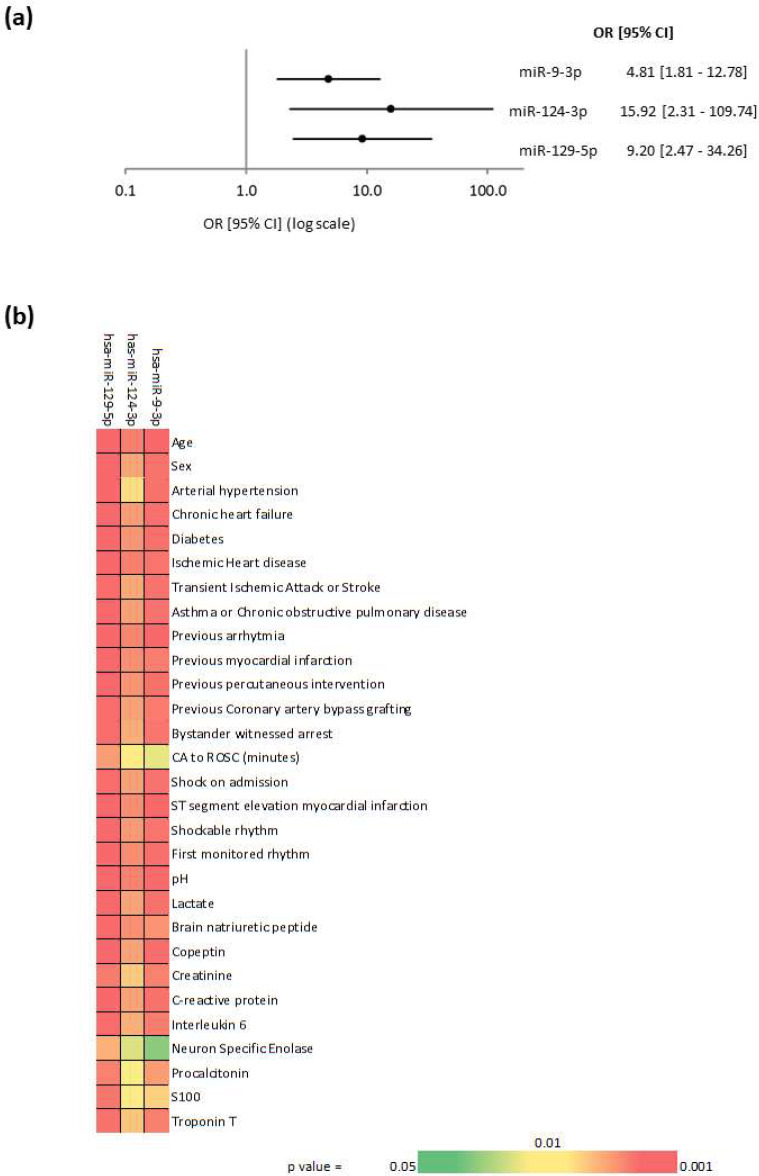
Association between miRNAs and neurological outcome. The association between levels of miR9-3p, miR124-3p and miR129-5p measured 48 h after ROSC in 50 patients and neurological outcome as attested by CPC score (1 vs. 5) at 6 months after cardiac arrest was addressed using univariate and multivariate logistic regression. (**a**) Forest plot with univariate odds ratios (OR) ± 95% confidence intervals (CI) for the prediction of neurological outcome. (**b**) Heat-map representing the statistical significance of multivariate logistic regression models containing one miRNA and one demographic or clinical variable. The coloured scale is as follows: *p* = 0.001 (red) and *p* = 0.05 (green), *p* = 0.01 being used as a middle point for the colour gradient. All values represented had a *p*-value below 0.05, and *p*-values below 0.001 were displayed as the maximum intensity of red.

Logistic regression was subsequently used to address a possible association between the three miRNAs and neurological outcome of the 50 patients, as determined by 6-month CPC. All three miRNAs were univariate predictors of neurological outcome (Figure 2a). This prediction was maintained (all *p* < 0.05) after adjustment with demographic and clinical variables (Figure 2b).

## 3. Discussion

This study was designed to address the correlation between circulating miRNAs and brain-injury in patients after cardiac arrest. We report strong correlations between the circulating levels of three brain-enriched miRNAs—miR9-3p, miR124-3p and miR129-5p—and NSE, suggesting that these miRNAs might reflect the extent of brain damage. In addition, these miRNAs were associated with neurological outcome and survival at 6 months. These observations support the value of brain-enriched miRNAs to aid in outcome prediction after cardiac arrest.

The correlation between miRNAs and NSE was only observed in patients with poor neurological outcome, i.e., in patients with severe brain injury. This observation strengthens the assumption that circulating levels of brain-enriched miRNAs reflect the extent of brain damage. The absence of significant correlation between miRNAs and comorbidities suggests that levels of miRNAs are not affected by the studied comorbidities, supporting a clinically relevant prognostic value, as shown in logistic regression analyses.

When adjusting with NSE in multivariate analyses, the statistical significance of each predictive model including miRNAs was reduced, especially for miR9-3p which was very close to the threshold for significance (*p* = 0.040), consistently with the strong correlation with NSE observed in Figure 1. Similarly, the prediction capacity of the miRNAs was reduced after adjustment with the time from cardiac arrest to ROSC, strengthening the correlation between circulating levels of miRNAs and brain damage. However, the adjustment did not cancel the predictive capacity of miRNAs, which supports an added predictive value of miRNAs to existing predictive variables and biomarkers in multimodal approaches, as previously reported [13,15].

MiR9-3p, miR124-3p and miR129-5p are involved in different physiological and/or pathological processes such as brain development, differentiation, plasticity and cancer [17,18,19,20,21]. Hence, their presence—or increased levels—in the blood after cardiac arrest most probably mirrors the disruption of the blood brain barrier and cellular dysfunction or death in the brain after cardiac arrest.

This short study has some limitations. The number of patients enrolled was limited to a subset of 50 patients selected from the Targeted Temperature Management (TTM) cohort who were enrolled in a short RNA sequencing experiment. This low patients’ number prevented adjusting for all demographic and clinical variables simultaneously in multivariate analysis, hence dampening the statistical power of the study. To avoid model overfitting, we conducted sequential adjustments with only one variable at a time. MiRNAs were measured in plasma samples while NSE was measured in the serum. However, miRNA levels are comparable in serum and plasma in these patients. The correlation between miRNAs and brain damage was assessed at a single time-point, 48 h after ROSC, which was chosen for consistency with previous studies [13,15,22] and because NSE reaches maximal levels at this time-point and achieves the highest prognostic value [3]. Some patients with co-morbidities may have died from other causes than brain injury which might impact the correlation between miRNAs and NSE. Angiography was performed in 66% of the studied patients at the discretion of the physician. An association between severe coronary stenosis and mortality cannot be ruled out. Lastly, the 50 patients enrolled in this study were treated at 33 °C and it would be interesting to address the correlation with NSE in patients treated at 36 °C, although NSE has not been shown to be significantly affected by target temperature [3].

## 4. Materials and Methods

### 4.1. Patients

A subgroup of patients from the Targeted Temperature Management trial (TTM-trial) was used in the present study. Detailed information about the TTM-trial’s design, recruitment, protocol and results has been published elsewhere [23,24]. The trial is registered at www.clinicaltrials.gov (NCT01020916) and was approved by ethical committees of the different participating countries. Informed consent was waived or obtained from each participant or relatives, according to the legislation in each country and in line with the declaration of Helsinki. The primary end-point of the TTM-trial was survival until end of trial and the main secondary outcome was neurological outcome at 6 months after cardiac arrest, as defined by the cerebral performance category (CPC) score. Patients with 6-month CPC scores of 1 or 2 were considered as having a good neurological outcome and patients with 6-month CPC scores of 3 to 5 were considered as having a poor neurological outcome [25].

For the present study, we considered a subset of fifty patients that exhibited either a good (CPC 1) or a poor neurological outcome or died (CPC 5) within the 6 months after cardiac arrest (25 patients per group). These two groups of patients have been used in a previous study and had similar demographic and clinical features than the entire TTM cohort [13]. The fifty patients received targeted therapeutic treatment at 33 °C.

### 4.2. Small RNA Sequencing

Small RNA sequencing was performed in the groups of 25 patients at Exiqon Services following in-house protocols. Detailed methods have been previously published [13]. Briefly, total RNA was extracted from 400 µL of plasma obtained 48 h after ROSC, digested with proteinase K, checked for quality, and used to prepare sequencing libraries. Libraries were subjected to single-end sequencing of 50 nucleotides fragments on the NextSeq 500 Illumina platform. Sequencing reads were mapped to miRBase version 20 containing 1871 human precursor sequences and 2772 human mature miRNAs [26]. Sequencing data were filtered, considering only miRNAs expressed with more than 5 counts in at least 12 samples of at least one of the outcome groups. MiRNA levels are expressed as number of transcripts per million reads.

### 4.3. Neuron-Specific Enolase

Measurements of NSE levels in serum samples obtained 48 h after ROSC have been conducted six months after completion of the trial in a core laboratory of the Centre Hospitalier de Luxembourg. Haemolysis was assessed and all samples had a haemolysis index below 500 ng/mL of haemoglobin. NSE levels were determined using a COBAS e601 apparatus with an Electro-Chemi-Luminescent-Immuno-Assay kit from Roche Diagnostics (Rotkreuz, Switzerland). The measuring range was 0.05–370 ng/mL and samples above the upper limit were diluted and reassessed. Assay sensitivity was 0.25 ng/mL and normal values were <17.0 ng/mL.

### 4.4. Statistical Analysis

Correlation analyses were performed using Spearman correlation for continuous variables and logistic regression for categorical variables. For continuous variables, a correlation coefficient below or above 0.6 was used as threshold for significance. For categorical variables, an adjusted *p*-value (Benjamini-Hochberg step-up false discovery rate) below 0.05 was used for statistical significance. For univariate and multivariate analyses, missing values were imputed using the R package MICE with 10 imputations. All skewed continuous variables were log2 transformed and scaled. Logistic regression was used to estimate the predictive value of the independent variables for neurological outcome. Univariate models were generated with each miRNA candidate. Multivariate models were generated with one miRNA and one clinical parameter at a time. The models were generated using the lrm function of the rms R package.

## 5. Conclusions

In conclusion, circulating levels of brain-enriched miRNAs are associated with NSE levels and hence reflect the extent of brain injury in patients after cardiac arrest. This observation strengthens the potential of brain-enriched miRNAs to aid in outcome prognostication after cardiac arrest.

## Figures and Tables

**Table 1:** Demographic and clinical characteristics of the study population.

Characteristic	ALL (*n* = 50)	Good (*n* = 25)	Poor (*n* = 25)	*p*-Value
**Age**	**63 (59–74)**	**62 (59–69)**	**70 (60–80)**	**0.039**
Sex	41 (82%)	21 (84%)	21 (84%)	1
COMORBIDITIES				
Arterial hypertension	21 (42%)	10 (40%)	11 (44%)	1
Chronic heart failure	3 (6%)	1 (4%)	2 (8%)	1
Diabetes	3 (6%)	1 (4%)	2 (8%)	1
Ischemic heart disease	11 (22%)	3 (12%)	8 (32%)	0.17
Transient ischemic attack or stroke	4 (8%)	2 (8%)	2 (8%)	1
Asthma or chronic obstructive pulmonary disease	5 (10%)	1 (4%)	4 (16%)	0.35
**Previous arrhythmia**	**11 (22%)**	**2 (8%)**	**9 (36%)**	**0.04**
Previous myocardial infarction	8 (16%)	2 (8%)	6 (24%)	0.25
ARREST CONDITIONS				
Bystander witnessed arrest	41 (82%)	22 (88%)	19 (76%)	0.46
Bystander cardiopulmonary resuscitation	37 (74%)	19 (76%)	18 (72%)	1
**Time from CA to ROSC (minutes)**	**22 (19–30)**	**20 (17–22)**	**30 (22–37)**	**0.001**
Shock on admission	8 (16%)	3 (12%)	5 (20%)	0.7
ST segment elevation myocardial infarction	29 (58%)	16 (64%)	13 (52%)	0.57
Shockable rhythm	46 (92%)	25 (100%)	21 (84%)	0.11
First monitored rhythm	44 (88%)	24 (96%)	20 (80%)	0.19
LABORATORY MEASUREMENTS				
pH	7.25 (7.15–7.32)	7.29 (7.18–7.33)	7.21 (7.13–7.29)	0.221
Lactate (mmol/L)	4.9 (2.9–8.7)	4.8 (3.1–7.2)	5 (2.9–9.5)	0.992
**Brain natriuretic peptide (NT-proBNP, pg/mL)**	**1856 (946–2932)**	**1327 (407–1872)**	**2324 (1494–4009)**	**0.005**
Copeptin (pmol/L)	48.74 (25.4–112.87)	53.43 (27.43–119.73)	31.63 (23.63–82.95)	0.491
**Creatinine (mg/dL)**	**0.99 (0.82–1.47)**	**0.9 (0.71–1.08)**	**1.33 (0.89–1.55)**	**0.007**
C-reactive protein (µg/mL)	143 (102–201)	142 (115–192)	144 (100–205)	0.961
Interleukin 6 (pg/mL)	166 (74–337)	120 (73–295)	196 (77–545)	0.332
**Neuron specific enolase (ng/mL)**	**27 (17–57)**	**20 (15–29)**	**60 (20–110)**	**0.003**
**Procalcitonin (µg/L)**	**1.11 (0.4–3.61)**	**0.64 (0.34–1.38)**	**3 (0.57–5.27)**	**0.015**
**S100 (S100A1B and S100BB, µg/L)**	**0.12 (0.07–0.21)**	**0.09 (0.07–0.14)**	**0.16 (0.1–0.26)**	**0.035**

Median (range) or number (percentage) are shown for continuous and categorical variables, respectively. Laboratory measurements were performed at 48 h after return of spontaneous circulation (ROSC) except for pH and lactate, which were measured at admission. Comparisons between good and poor outcome have been performed using the Wilcoxon signed-rank test for continuous variables or the Fisher exact test for categorical variables. *p*-values < 0.05 are considered significant and are in bold. CA: cardiac arrest; ROSC; return of spontaneous circulation.

## Abbreviations

CA	Cardiac arrest
CI	Confidence intervals
CPC	Cerebral Performance Category
miRNAs	MicroRNAs
NSE	Neuron-specific enolase
OR	Odds ratio
ROSC	Return of spontaneous circulation
TTM	Targeted Temperature Management trial
